# Suicidal Erythrocyte Death in Metabolic Syndrome

**DOI:** 10.3390/antiox10020154

**Published:** 2021-01-21

**Authors:** Ignazio Restivo, Alessandro Attanzio, Luisa Tesoriere, Mario Allegra

**Affiliations:** Dipartimento di Scienze e Tecnologie Biologiche, Chimiche e Farmaceutiche Università di Palermo, 90128 Palermo, Italy; ignazio.restivo@unipa.it (I.R.); alessandro.attanzio@unipa.it (A.A.)

**Keywords:** eryptosis, metabolic syndrome, diabetes, dyslipidemia, hypertension, obesity, atherosclerosis, vascular damage, oxidative stress, endothelial dysfunction

## Abstract

Eryptosis is a coordinated, programmed cell death culminating with the disposal of cells without disruption of the cell membrane and the release of endocellular oxidative and pro-inflammatory milieu. While providing a convenient form of death for erythrocytes, dysregulated eryptosis may result in a series of detrimental and harmful pathological consequences highly related to the endothelial dysfunction (ED). Metabolic syndrome (MetS) is described as a cluster of cardiometabolic factors (hyperglycemia, dyslipidemia, hypertension and obesity) that increases the risk of cardiovascular complications such as those related to diabetes and atherosclerosis. In the light of the crucial role exerted by the eryptotic process in the ED, the focus of the present review is to report and discuss the involvement of eryptosis within MetS, where vascular complications are utterly relevant. Current knowledge on the mechanisms leading to eryptosis in MetS-related conditions (hyperglycemia, dyslipidemia, hypertension and obesity) will be analyzed. Moreover, clinical evidence supporting or proposing a role for eryptosis in the ED, associated to MetS cardiovascular complications, will be discussed.

## 1. Eryptosis

### 1.1. Mechanisms

The lifespan of erythrocytes (RBC) is limited by senescence [1,2]. Within 100–120 days, aged RBC are cleared from circulation through a mechanism involving the clustering and/or breakdown of the anion exchanger protein band 3 (AE1). This is regarded as the central step of an immune-mediated pathway, eventually leading to the disruption of the AE1-dependent cytoskeletal connections to the lipid bilayer. The process, then, culminates with vesicle generation, volume, density and shape changes and senescent antigens uncovering [2].

During the course of their natural ageing and prior to senescence, RBC may experience injury that weakens their integrity, function and survival. Under these circumstances, RBC undergo hemolysis that implies the rupture of the cell membrane and the release of hemoglobin (Hb). Should this happened, Hb is filtered by renal glomeruli and subsequently precipitates in the acidic lumen of renal tubules, eventually leading to renal failure [3,4].

Alternatively, RBC may enter a suicidal death program named eryptosis [5]. Similar to the apoptotic death of nucleated cells, eryptosis is a coordinated, programmed cell death eventually culminating with the disposal of cells without disruption of the cell membrane and release of intracellular oxidative and pro-inflammatory milieu [6]. Along these lines, eryptosis can be regarded as a “soft” mechanism removing, prior to hemolysis, defective, infected or otherwise potentially deleterious RBC [7].

The hallmarks of eryptosis are similar to those of apoptosis i.e., cell shrinkage, membrane blebbing and exposure of phosphatidylserine (PS) on the cell membrane. The process can be triggered by several physio-pathological cell stressors, such as hypertonic shock, energy deprivation, increased temperature and oxidative stress [4,8]. Moreover, a number of xenobiotics, heavy metals, drugs and physiological mediators can also be involved in the activation of the process [8,9]. 

**Figure 1:**
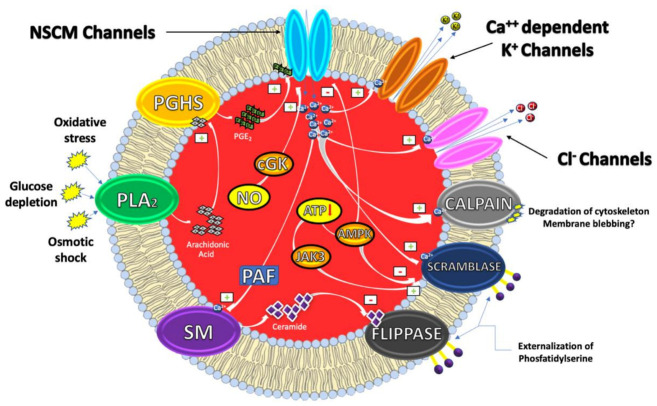
Eryptosis signaling.

From a mechanistic perspective, eryptosis is orchestrated by an amazingly complex cellular machinery involving Ca^++^, reactive oxygen and nitrogen species (RONS), ceramide, caspases, nitric oxide (NO) and a variety of kinases (Figure 1).

It has, indeed, been demonstrated that cytosolic Ca^2+^ concentrations can play a pivotal role in the eryptotic process triggered by oxidative stress, glucose depletion and osmotic shock. Under these conditions, the pro-eryptotic stimuli activates phospholipase A_2_ (PLA_2_) that releases arachidonic acid (AA) in turn metabolized by prostaglandin endoperoxide synthase (PGHS). The resulting increase of prostaglandin E2 (PGE_2_) synthesis then activates and opens nonselective cationic membrane (NSCM) channels, leading to an increase of endocellular levels of Ca^++^ that activates several molecular targets including Ca^++^-dependent K^+^ channels. The subsequent membrane hyperpolarization, increases the electrical driving force for Cl^−^ exit. The resulting cellular loss of KCl, with osmotically obliged water efflux, determines RBC shrinkage [9,10]. On the other hand, Ca^++^ influx, together with platelet activating factor (PAF), activates membrane sphingomyelinase (SM) responsible for the increase of ceramide levels that plays a crucial role in eryptosis. Indeed, Ca^++^ and ceramide, respectively, activate scramblase and inhibit flippases involved in PS externalization. Finally, an increase of Ca^++^ levels also stimulates cysteine-calpain endopeptidases, responsible for the degradation of RBC cytoskeleton and for membrane blebbing that increases the adhesiveness of RBC. Once the exposure of PS takes place, RBC is recognized by circulating macrophages with specific PS receptors and engulfed to ensure its effective removal from circulation [4,11].

Beside Ca^++^ and/or ceramide increase, other signaling pathways, involving kinases, NO and caspases further contribute to modulate the eryptotic process. Indeed, in RBC subjected to energy depletion or oxidative stress, Janus-activated kinase 3 (JAK3) can be phosphorylated, activated and stimulates cell membrane scrambling. On the other hand, hyperthermia or energy deprivation can activate the energy sensing AMP-activated kinase 1 α (AMPK1 α) that inhibits eryptosis. Furthermore, during oxidative stress conditions, eryptosis is also stimulated through the activation of casein kinase 1 α (CK1 α) while it is further inhibited by cGMP-dependent protein kinase (cGKI). This latter can be stimulated by NO, usually stored in RBC, and released upon Hb deoxygenation [9,12].

Interestingly, NO together with NO-donors such as nitroprusside, have been demonstrated to inhibit Ca^++^-induced eryptosis. The mechanism through which NO interferes with the Ca^++^ signaling seems to involve downstream mediators without affecting Ca^++^ levels and involving caspases [13].

As in nucleated cells, caspases are expressed in RBC, where they cleave the AE1 and stimulate PS exposure. However, in contrast to apoptosis of nucleated cells, they do not always play a dominant role in the eryptotic process. Caspase activation is, indeed, involved in the eryptosis induced by leukotrienes and α-lipoic acid and fostered by oxidative stress. Conversely, Ca^++^ entry and Ca^++^-dependent cell membrane scrambling do not require the activation of caspases [9,12].

While providing a convenient form of death for RBC (by counteracting hemolysis and its complications), dysregulated eryptosis may result in a series of detrimental and harmful pathological consequences. In this regard, it has been shown that eryptosis underlies and fosters several different clinical conditions or diseases such as anemias, cytostatic-induced malignancies, sepsis, psychosis and malaria [8,12,13].

### 1.2. The Impact of Eryptosis on Endothelial Cells and Thrombocytes

Due to tendency of eryptotic RBC to adhere to endothelial cells (EC), and to dysfunctionate them, eryptosis is also involved in the pathogenesis of inflammatory-related cardiometabolic diseases [3,12]. PS-exposing RBC have, indeed, been demonstrated to exhibit an increased adhesiveness to the vascular wall, mainly through the binding between PS and endothelial CXC-Motiv-Chemokin-16/Scavenger Receptor for PS and Oxidized Low Density Lipoprotein (CXCL16/SR-PSOX) [14]. Coherently, endothelial CXCL16 exerts a crucial role in leukocyte adhesion to endothelium both in experimental set-ups and at the sites of atherosclerotic lesions [15]. Furthermore, PS-exposing RBC can also bind (although to a lesser extent,) additional structures such as the heparin-binding domain of endothelial or subendothelial thrombospondin-1 (TSP-1) and endothelial PS receptors CD36 [16,17,18].

Interestingly enough, not only can PS-exposing RBC adhere to EC but also stick to blood platelets by binding CXCL16/SR-PSOX and/or CD36. Hyper-adhesiveness of eryptotic RBC to thrombocytes compromises microcirculation, fosters blood clotting and, as a result, increases the risk of thrombosis [19,20]. Eryptosis-dependent RBC cross-talk with thrombocytes, therefore, translates vascular inflammation into thrombo-occlusive events in different diseases [20,21].

In the light of the crucial role exerted by the eryptotic process in the endothelial dysfunction (ED), the focus of the present review is to report and discuss on the involvement of eryptosis within a cluster of cardiometabolic factors, i.e., the Metabolic Syndrome (MetS), in which vascular complications are utterly relevant.

Current knowledge on the mechanisms leading to eryptosis in MetS-related conditions (hyperglycemia, dyslipidemia, hypertension and obesity) will be analyzed. Moreover, clinical evidence supporting or proposing a role for eryptosis in the ED associated to MetS cardiovascular complications (especially those associated with diabetes and atherosclerosis) will be discussed.

## 2. The Metabolic Syndrome

According to its “harmonized” definition, MetS is described as a cluster of metabolic factors that increases the risk of cardiovascular diseases, diabetes (DM) and associated morbidities such as dementia [22,23]. More specifically, the combined occurrence of at least three of the following five risk factors would qualify a person for the MetS: hyperglycemia, hypertriglyceridemia, low High-Density Lipoprotein (HDL) cholesterol levels, hypertension and abdominal obesity (Table 1) [23].

MetS can be modulated by genetics and epigenetics factors, fostered by a sedentary lifestyle, influenced by the quality of both food and sleep and controlled by gut microbiota [23]. It has been estimated that more than 33% in the United States, and approximately 40% of people over the age of 40, have MetS, making it a major health hazard of the modern world [24].

From a mechanistic perspective, MetS development strongly depends on the establishment of a low-grade, systemic, chronic inflammation. Indeed, the dysfunctional activation of the inflammatory response strongly impairs the metabolic homeostasis of key tissues in energy utilization such as liver, adipose, muscle and intestine [25,26]. Along these lines, a pro-inflammatory, pro-oxidant and pro-thrombotic state has been observed in MetS patients [27,28]. Fat accumulation and obesity are major players in the development of chronic inflammation, due to the innate capacity of adipocytes to secrete pro-inflammatory mediators such as adipocyte-derived cytokines as well as hormones such as leptin in response to hypertrophic signals [29,30,31]. Moreover, hypertrophic adipocytes also increase the recruitment, retention and activation (within the adipose tissue itself) of the pro-inflammatory M1-type macrophages, responsible for the release of proinflammatory mediators (IL-1β, IL-6 and TNF-α) in the bloodstream [32,33].

This event, in turn, favors an increased efflux, from adipocytes, of free fatty acids (FFA) and their ectopic deposition (lipotoxicity) in the liver and the skeletal muscle [34,35,36,37]. Here, the resulting convergence of pro-inflammatory and lipotoxic mediators, with FFA playing a major role, inhibits insulin signaling. Eventually, the process culminates with the establishment of an insulin resistance state, accompanied with hyperglycemia and subsequent hyperinsulinemia [26,32].

Moreover, in susceptible individuals, the inability of the pancreas to compensate for insulin resistance results in a condition of relative hypo-insulinemia that further increases the lipolysis of stored triglycerides (TG) and release of FFA [38]. These latter are, in turn, vehiculated into the portal circulation, shuttled to the liver, reassembled as VLDL and released in the bloodstream, resulting in hypertriglyceridemia [32,39].

Relevantly, this condition does also affect cholesterol metabolism. Indeed, the exchange of TG (deriving from VLDL) for cholesterol esters (from HDL) results in a rapid clearance of HDL. At the same time TG in excess are also transferred to LDL, which then becomes a more attractive substrate for hepatic lipase and, after lipolysis, they are transformed into small dense LDL [38,39]. These particles are more atherogenic than larger LDL subclasses as they are more prone to oxidation and uptake into the arterial wall [32].

As a consequence of hepatic insulin resistance and abundance of FFA substrates, gluconeogenesis is increased and fosters hyperglycemia. Myocellular insulin resistance also results in decreased glucose disposal peripherally. Over time, pancreatic β-cells persist in decompensating for the increased need for insulin to overcome resistance, leading to the establishment of a DM condition [23,36].

Impaired glucose homeostasis, in turn, exacerbates inflammation and tissue damage, thus sustaining and reinforcing a pro-inflammatory state eventually leading to the ED [30].

ED is a pathological state resulting from an imbalance between vasoconstrictor and vasodilator responses, leading to a dysfunctional vascular tone, increased peripheral vascular resistance and reduced organ perfusion. It is one of the earliest events in the development of atherosclerotic lesions and a landmark of MetS itself. ED is strictly related to oxidative stress and inflammation via the activation of several redox-dependent transcription factors such as nuclear factor kappa-B (NF-κB) [1].

ED has been shown to play a key role in the development of a major risk factor associated with MetS i.e., hypertension. Along these lines, increased levels of blood pressure in MetS patients have been correlated with the ED generated by the FFA-induced RONS increase. MetS-related hypertension can be also fostered by the hyperinsulinemia-mediated activation of the sympathetic nervous system and through the inhibition of nitric oxide synthase (NOS). Moreover, adipose tissue-derived cytokines and the obesity-dependent hyperactivity of the renin-angiotensin-aldosterone system are also involved in the etio-pathogenesis of MetS-related hypertension [40,41].

In the following sections we will review the key role exerted by eryptosis in the development of MetS-associated risk factors (hyperglycemia, dyslipidemia, hypertension and abdominal obesity) and clinical complications (DM and atherosclerosis).

## 3. Eryptosis, Hyperglycemia and Diabetes

DM is a group of metabolic disorders hallmarked by a long-standing hyperglycemia, oxidative stress and reduced secretion and/or efficacy of insulin. All these metabolic dysregulations eventually lead to vascular complications that may enhance the risk of stroke, heart and kidney diseases [42]. Retinopathy, nephropathy, neuropathy and atherosclerosis are, indeed, among the most serious DM long-term complications [42,43] and make DM an alarming clinical and public health problem [44,45]. The International Diabetes Federation estimates that just under half a billion people are living with DM worldwide and the number is projected to increase by 25% in 2030 and by 51% in 2045 [46].

RBC lifespan has been shown to be reduced in DM patients [47]. In agreement, anemia is prevalent in 14–45% of DM-affected subjects and cannot be attributed to a decreased RBC formation, as reticulocyte number is increased [48,49]. Instead, increasing evidence has revealed that DM-related anemia may result, at least in part, from an enhanced eryptosis [50,51].

From a mechanistic perspective, DM-induced eryptosis has been related to glycoxidation, an oxidative stress-dependent glycation of biomolecules regarded as a DM hallmark [52,53,54]. This process, via hyperglycemia-mediated and oxidative stress-dependent mechanisms, leads to a dysfunctional alteration of RBC morphology and culminates with the increase of PS levels on cell surface [8].

According to the glycoxidation hypothesis, a long-lasting increase of blood glucose levels accelerates the glycation of free amino groups in RBC membrane proteins. Such non-enzymatic modification of proteins leads to the formation and accumulation of both early and advanced glycation end-products, EGEs and AGEs, respectively [55,56,57]. The latter mediate cross-linking of cellular biomolecules, eventually leading to the accumulation of misfolded, aggregated and nonfunctional proteins. When hyperglycemia is associated with oxidative stress, less reactive EGEs are increasingly been converted into higher reactive AGEs. Among the AGEs, carboxymethyl-lysine (CML), carboxyethyl-lysine (CEL) and methylglyoxal (MG) play the major role in DM.

CML and CEL induce insulin resistance, β-cell dysfunction, vascular toxicity and are involved in the development of diabetic complications [57,58,59]. Relevantly, they have been recovered in DM patients at a plasma concentration of 4.9 and 1.7 μM, respectively [60,61].

In addition to their effects on the liver, pancreas, muscle and EC, a novel physio-pathological role for both CML and CEL has recently emerged. Indeed, these AGEs have been shown to induce eryptosis-related morphological changes in RBC that completely lose their normal discoid shape and become acanthocytes [55]. The mechanisms through which CML and CEL promote these dysfunctional and morphological RBC modifications seems to be related to the pro-oxidant nature of these compounds and mediated by cytoskeleton modifications.

MG derives from the metabolism of sugars, amino acids and lipids and is able to react with free arginine residues of proteins forming Arg-pyrimidine adducts [62]. Interestingly, this dicarbonylic compound has gained significance in the etio-pathogenesis of DM, being able to impair insulin signaling and mediate DM-related vascular complications [63,64]. Relevantly, in vivo studies clearly show an increase of MG concentrations in the plasma of DM patients, in a range between 0.3 and 0.5 µM [65,66,67,68].

Along with CML and CEL, MG can also activate the eryptotic program at concentrations of physio-pathological relevance [69]. Interestingly enough, not only has MG been recovered in the plasma of DM patients, but also it can be generated inside RBC. In vitro evidence clearly showed that MG levels are increased in RBC exposed to high-glucose levels in response to the increase in the triosephosphate pool of glycolytic intermediates [70,71].

From a mechanistic perspective the pro-eryptotic role of MG may be connected with its ability to induce energy depletion and oxidative stress without involving alterations of Ca^++^ homeostasis [69]. Indeed, MG can interfere with glyceraldehyde-3-phosphate dehydrogenase, inhibiting glycolysis and thus decreasing ATP intracellular concentrations. The resulting energy depletion may, in turn, compromise GSH synthesis, thus interfering with anti-oxidative defense. Relevantly, DM patients exhibit a significant reduction of GSH plasma concentration and, interestingly, this condition positively correlates with diabetic complications. Moreover, and besides its effect on GSH levels, MG can also impair RBC antioxidative defenses, inactivating glutathione peroxidase (GPx). Other mechanisms could support MG-induced eryptosis, such as crosslinking of matrix proteins or glycosylation of plasma proteins [69].

Beside specific, AGEs-related pro-eryptotic mechanisms, other crucial molecular details have been unveiled in the activation of the DM-induced, RBC suicidal death program.

In contrast with other pro-eryptotic stimuli, DM induces eryptosis through a mechanism that appears to be independent of intracellular Ca^++^ variations. In this regard, an inhibition of the cation channel, possibly related to the employment of Ca^++^ channel blocking drugs by DM patients, has been suggested. Moreover, the lack of increased intracellular Ca^++^ concentration could explain the absence of cell shrinkage, in contrast to the majority of eryptotic stimulators that decreases RBC volume through the activation of Ca^++^-sensitive K^+^ channels [72].

The activation of eryptosis in DM has also been related to intracellular ceramide levels [72]. The amount of this lipid signaling molecule is significantly upregulated in RBC from DM patients. What is noteworthy, is that it correlates with the percentage of PS exposure, suggesting it could be an important candidate in the pathogenesis of DM-related eryptosis [72].

As previously stated, caspase-3 activation is a key element in the eryptotic process triggered by a number of conditions. In line with this, in vivo studies showed that RBC from DM patients undergo eryptosis through the activation of caspase-3, which is strongly correlated to the extent of hyperglycemia and extracellular oxidative stress. Caspase-3 activation can be considered as an effector mechanism contributing, together with other molecular machineries, to the reduction of diabetic RBC lifespan [51].

Mechanistic evaluations of DM-related eryptosis should also consider the therapeutic approach of DM patient. Indeed, a wide variety of xenobiotics and drugs can influence RBC morphology and PS-exposure [4]. Eryptosis, thus, can also be modulated by treatment of the patients, e.g., with the use of Ca^++^ channel blockers or antioxidants. Moreover, diabetic complications, such as nephropathy, dehydration, iron deficiency and inflammation, may also contribute to the activation of eryptosis [4].

Beyond mechanistic investigations and in the light of the ability of eryptotic RBC to induce ED, another key issue is to establish whether eryptosis can exert a pathophysiological role in DM-related vascular complications.

These life-threatening events associated with DM are deeply dependent on glycoxidation-induced microcirculation impairment and ED [42,54]. Relevantly, ex vivo evidence shows that enhanced PS exposure on eryptotic RBC from DM patients fosters RBC adhesion to the EC [73]. It has been suggested that the loss of lipid asymmetry could be responsible for the increased adhesiveness of RBC to vascular endothelium and for their ability to impair microcirculation [73]. Moreover, clinical data suggest that DM-induced eryptosis, through the impairment of RBC deformability induced by caspase-3 activation, could contribute to the pathogenesis of the hypertensive complications of DM patients [51]. Overall, ex vivo and in vivo evidence could support a role for eryptosis in the development of ED associated to DM-related vascular complications. Additional experimental effort is required to gain more mechanistic insights and to further characterize its clinical relevance.

Besides its pathological consequences, the activation of the eryptotic process in DM also deserves particular attention from a diagnostic perspective.

Glycoxidation-mediated reduction of the RBC lifetime may, indeed, affect the interpretation of glycated Hb (HbA1c) concentration, which is widely used to monitor metabolic control in diabetic patients. In fact, the degree of Hb glycation strongly depends on both blood glucose concentration and time of exposure. RBC life span negatively correlates with glycemia and averages ~80 days in patients with poor metabolic control as compared to the normal range of 123 days [32]. Accordingly, the resulting HbA1c value maybe lower in diabetic patients with high plasma levels of AGEs and could not fully mirror an eventual poor metabolic control.

## 4. Eryptosis, Dyslipidemia and Atherosclerosis

Dyslipidemia of MetS patients results from the concerted action of insulin resistance and obesity and has recently been described as “metabolic dyslipidemia” [74]. Clinically, it is shown as hypertriglyceridemia with low HDL plasma levels and increased small dense LDL/LDL ratio. According to data from 2009 to 2012, such a condition affects more than 100 million U.S. adults, aged 20 years or older [75].

Over the last years, a novel modulatory role for RBC in the development of dyslipidemia-induced cardiovascular complications, such as those related to atherosclerosis, has emerged. RBC, indeed, can become entrapped within atherosclerotic lesions at sites of intraplaque hemorrhage. Here they are actively taken up by macrophages, with the extent of RBC extravasation and foam cell formation, proportional to plaque development [76,77,78]. Along these lines, the involvement of eryptosis in dyslipidemia-induced vascular complications has started to gain researchers’ attention.

Clinical evidence shows that RBC from dyslipidemic patients are characterized by a grade of eryptosis significantly higher than healthy subjects [79]. The mechanisms, underlying the activation of the eryptotic program in such patients, do not involve any alteration of Ca^++^ homeostasis. Rather, the increased levels of PS externalization on cell membrane appear to be dependent on endocellular oxidative stress. Coherently, RBC form dyslipidemic patients show reduced amount of plasma GSH and increased levels of lipid peroxidation markers, with respect to healthy subjects [79].

The oxidative stress-dependent increase of PS-externalization on RBC membrane has also been investigated in a mice model of high-fat diet (HFD)-induced dyslipidemia [80].

Interestingly, this model does provide several relevant mechanistic insights on the involvement of eryptosis in the proatherogenic effects of HFD with specific regard to the key role exerted by the cross-talk between eryptotic RBC, macrophages and EC in the development of the atherosclerotic plaque.

Interestingly, in their paper, authors show that HFD-dependent increase of PS externalization on RBC fosters their pro-inflammatory interactions with macrophages. In addition, the resulting phagocytosis of HFD-RBC induces a pro-inflammatory shift of the macrophage phenotype that promotes macrophage-EC interactions. Moreover, not only did it result in HFD-RBC in increased interactions with macrophages but also in the activation of luminal endothelium and in the consequent increase of monocyte binding. This may eventually fuel endothelial inflammation, thereby enhancing the development of atherosclerosis.

The HFD-induced increased erythrophagocytosis and EC dysfunction could provide an additional, eryptosis-dependent mechanism for the pro-atherogenic role of RBC. Further in vivo, ex vivo and in vitro studies would be needed to elucidate whether and how dyslipidemia-induced eryptosis could exert a significant role in triggering vascular inflammation, contributing to atherogenesis.

Dyslipidemia-induced cardiovascular complications strongly involve oxidative stress [81,82]. Along these lines, both cholesterol and fatty acids oxidation by-products have been found in plasma and atherosclerotic lesions of dyslipidemic patients [83,84,85,86].

Oxysterols are cholesterol oxidation by-products entrapped in circulating LDL. They are able to generate ROS through the up-regulation of NADPH oxidase (NOX) family enzymes [87]. Relevantly, oxysterols are deeply involved in ED making EC more susceptible to stress stimuli and to cardiovascular pathogenesis. Furthermore, 7-ketocholesterol (7-KC), cholestan-3β,5α,6β-triol (TRIOL), 5α,6α- and 5β,6β-epoxy-cholesterol and 27-hydroxycholecholesterol are the most abundant oxysterols in the plasma and atherosclerotic lesions [88].

Interestingly, in vitro and ex vivo evidence shows that a mixture of oxysterols, qualitatively and quantitatively consistent with the oxysterol pool in the plasma of hypercholesterolemic subjects (20 µM total oxysterols), induces eryptosis in healthy human RBC [89]. The mechanism underlying the oxysterol-induced eryptosis, is mediated by PGE_2_ release and by the opening of PGE_2_-dependent Ca^++^ channels. Not only involves it a partial alteration of Ca^++^ homeostasis but also RONS production, GSH depletion and membrane lipid peroxidation. The activation of Ca^++^-independent PKC and caspase-3, as well as the inhibition of the amino-phospholipid translocase-mediated inward transport of PS are also involved in the pro-eryptotic activity of oxysterols. Interestingly enough, among all the oxysterols individually-tested, only 7-KC and TRIOL are able to exert an eryptotic activity.

The redox-dependent, pro-eryptotic mechanism of these compounds strongly depends on the RBC RONS-generating enzymes: RBC-NOX, RBC-NOS and xanthine oxidoreductase (XOR) [90]. More specifically, RBC-NOX is the target of 7-KC, through the activation of Rac GTPase and PKCζ pathway. On the other hand, TRIOL activates RBC-NOS via the PI3K/Akt axis, with the contribution of a Rac-GTPase. Along with the TRIOL-induced NO production, metHb formation and heme loss have been observed and attributed to nitrosative stress. Redox modifications of the RBC milieu by either RBC-NOX or RBC-NOS, in turn activate XOR reinforcing the overall oxidative/nitrosative stress by either oxysterols. Relevantly, when combined, 7-KC and TRIOL act independently. Their effects on RONS production and PS exposure appear as the result of the effects of the oxysterols on RBC-NOX and RBC-NOS.

Within fatty acids by-products, 4-hydroxy-trans-2-nonenal (HNE) has gained significance in the etio-pathogenesis of several, inflammatory-based dysmetabolic conditions [85]. HNE is an α, β-unsaturated aldehyde, endogenously generated by the radical-mediated peroxidation of ω-6 polyunsaturated fatty acids [91]. Thanks to its electrophilic moieties it can covalently bind to biomolecules, modifying them. It has been shown that plasma levels of HNE, in the range between 10 and 100 µM, constitute one of the main driving forces in the development of insulin resistance and dyslipidemia-dependent cardiovascular complications [86].

Along these lines, recent in vitro evidence has shown that HNE at physiopathological concentrations can induce eryptosis in human healthy RBC [92]. The mechanism responsible for such activity is mediated by PGE_2_ release and by the opening of PGE_2_-dependent calcium channels. Differently from the oxysterol-induced eryptosis, the HNE-mediated one does not depend on oxidative stress. Rather, it involves alteration of Ca^++^ homeostasis, activation of caspase-3, as well increase of ceramide levels. Moreover, HNE-activated RBC show an altered, eryptotic morphology and the presence of agglutination elements through which RBC could interact with platelets and/or EC. Coherently, PS externalization on RBC membrane fosters RBC interaction with EC. Relevantly, HNE-induced, eryptosis mediated, RBC hyper-adhesiveness eventually result in EC dysfunction as evaluated by ICAM-1 overexpression.

Collectively, in vitro and ex vivo data suggest new and additional mechanisms through which the cholesterol and fatty acid by-products could contribute to vascular dysfunctions characteristic of MetS. Moreover, clinical and in vivo evidence coherently points out that eryptosis could provide an additional mechanism through which RBC could contribute to the development of atherosclerosis and other dyslipidemia-related complications. These mechanisms might well be dependent on the resulting, eryptosis-dependent hyper-adhesiveness of RBC to EC, macrophages and platelets.

## 5. Eryptosis and Hypertension

Arterial hypertension (AH), a condition hallmarked by increased arterial pressure, either diastolic or systolic, frequently occurs in patients affected by MetS. AH-induced cardiovascular and renal complications can also be worsened by other conditions, such as smoking, alcoholism, psychological stress, diabetes mellitus, or dyslipidemia [93,94]. According to the World Health Organization, one out of three adults in the world suffers from AH [95]. ED does play a crucial role in the pathogenesis of AH, being EC the biological interface where proinflammatory and pro-oxidative signals converge [96].

Recent clinical evidence demonstrates that RBC from hypertensive patients are characterized by high levels of eryptosis as evaluated by PS externalization on cell membrane [79]. The mechanisms underlying AH-induced eryptosis are mediated by an increase of both intracellular Ca^++^ concentration and oxidative stress. Indeed, RBC from hypertensive patients show higher levels of lipid peroxidation markers than normotensive individuals while leukocyte exhibit increased amount of superoxide ions, NO and hydrogen peroxide. In addition, RBC from AH patients are characterized by reduced activities of several antioxidative enzymes, including GPx, catalase and superoxide dismutase, as well as lower GSH levels as compared to normotensive individuals [79].

In the light of this clinical evidence, further ex-vivo studies are warranted to elucidate more in depth the molecular mechanisms underlying the AH-induced eryptosis. Moreover, additional clinical and in vivo data are needed to unveil if, and to what extent, eryptosis could play a role in the AH-dependent ED associated with MetS vascular complications.

## 6. Eryptosis and Obesity

Notwithstanding almost 30% of obese individuals are free of metabolic co-morbidities (the so-called “metabolically healthy obese”), obesity represents a crucial risk factor for oncological, neurodegenerative and cardiometabolic diseases [27,97,98].

Interestingly, recent evidence links obesity to an increased risk of thrombosis [99]. In this regard, a key role in the development of thrombotic events has emerged for hemorheological properties such as RBC aggregability (EA) and deformability (ED).

Along these lines, an interesting in vivo study has showed that subjects with a higher body mass index exhibit high levels of PS exposure on RBC membrane and increased EA index. These data suggest that eryptosis could contribute to hypercoagulability in obese patients [100]. From a mechanistic perspective, obesity-induced eryptosis is dependent on RBC oxidative stress as the subjects with a higher body mass index also display increased MDA levels compared to control ones. Relevantly, obesity-stimulated eryptosis is reversible, as PS levels on RBC membrane decreased when the subjects enrolled in the study went on a diet [100].

## 7. Conclusions

The role of RBC in the establishment of MetS-related cardiovascular diseases is well established [101]. The eryptosis involvement in MetS and its clinical complications reveals novel, significant and additional pathways through which RBC could participate, via oxidative stress-dependent mechanisms, in the development of MetS-associated vascular complications.

## Figures and Tables

**Table 1:** Criteria for clinical diagnosis of metabolic syndrome (MetS) [23].

Parameters	Cut-Points
Waist Circumference	Population- and Country-specific Definitions
Triacylglycerols	≥150 mg/dL
HDL Cholesterol	Males: ≤40 mg/dL Females: ≤50 mg/dL
Blood Pressure	Systolic: ≥130 and/or Diastolic: ≥85 mmHg
Fasting Plasma Glucose	≥100 mg/dL
